# On the origin of our fascination with crystals

**DOI:** 10.3389/fpsyg.2026.1633599

**Published:** 2026-03-04

**Authors:** Juan Manuel García-Ruiz, Tomás de la Rosa, Irene Delval, Guillermo Bustelo

**Affiliations:** 1 Donostia International Physics Center, San Sebastián, Spain; 2 Neuropsychopharmacology & Psychobiology Research Group, Department of Neuroscience, University of Cádiz, Cádiz, Spain; 3 Centro de Investigación Biomédica en Red en Salud Mental (CIBERSAM), Instituto de Salud Carlos III, Madrid, Spain; 4 Instituto de Investigación e Innovación Biomédica de Cádiz (INiBICA), Cádiz, Spain; 5 Instituto de Psicologia, Universidade de São Paulo (IPUSP), São Paulo, SP, Brazil; 6 Rainfer, Madrid, Spain

**Keywords:** chimpanzee, cognition, crystal, evolution, material engagement theory, *Pan troglodytes*, quartz

## Abstract

Crystals are fascinating structures of solid or liquid matter where atoms, molecules, and/or ions are, on average, arranged in a highly ordered lattice. It is well-documented that some of the earliest objects collected by our hominin ancestors, without evident practical purpose, were small quartz and calcite crystals. These crystals, measuring a few centimeters, had no known utility as weapons, tools, or ornaments. However, hominins appear to have appreciated these stones, collecting and transporting them from their place of discovery to their shelters. This behavior, registered as far back as 780,000 years ago, has been interpreted as early evidence of symbolic thought in much younger archaeological contexts. In this study, we adopted an experimental approach to investigate the factors underlying the nature of our ancestral fascination with crystals. We designed a series of experiments with enculturated chimpanzees, one of our two closest living nonhuman relatives, who share significant genetic and behavioral traits with humans. The experiments aimed to identify which physical properties of crystals might have attracted chimpanzees and hominins. Our results suggest that enculturated chimpanzees can identify and distinguish crystals from other types of stones. We found that transparency and geometric shape were the two attractors guiding chimpanzees. These properties are notably salient in the natural environments of both chimpanzees and hominins. Furthermore, the crystals elicited exploratory behaviors in the enculturated chimpanzees, who engaged in voluntary and intentional actions to investigate crystalline transparency and compare shape. We discuss the relevance of these findings for understanding hominin behavior, proposing that similar responses to crystals in hominins and non-hominin apes could reflect a shared cognitive predisposition. Our study provides insights into the potential role of crystal collection in cognitive evolution and highlights the significance of material properties in shaping early symbolic behaviors.

## Introduction

The definition of crystal has changed over time. Since 2021, the International Union of Crystallography (IUCr) has defined a crystal based on the diffraction pattern of its internal ordered structure (Grimm, 2015; Amorós, 2017). However, historically, crystals were described in terms of their external morphologies, characterized by the angular and symmetry relationships between their faces leading, in ca. 1800, to the Law of Rational Indices (Amorós, 2017). Before the discovery of the internal order of crystals at the end of the 18th century (Amorós, 2017), the most distinctive feature of a crystal was its singular morphology, which was different from any other solid natural object. A second astonishing feature of quartz crystals is their transparency. Indeed, the word *crystal* comes from the Greek “cryos” and, etymologically, means supercooled water. The classic Greeks thought that transparent crystals of quartz, commonly known as rock crystals, were water that had become so solid that it was impossible to melt it again.

Archeological evidence strongly suggests that crystals were among the first natural objects collected by hominins without any apparent utilitarian purpose (Bednarik, 2003; Harrod, 2014; Wilkins et al., 2021). In 1931, Wenzhong Pei reported the discovery of twenty quartz crystals in the renowned Zhoukoudian site, alongside *Homo erectus* remains dated Lower Pleistocene (Pei, 1931) and more recently dated at least 600 ka and possibly >800 ka (Shen et al., 2001). Additional findings have since corroborated this behavior. In 1989, at Singi Talav site in India, six nearly complete quartz prisms were found in a stratum from the Lower Acheulian (between 300,000 and 150,000 years old) (d’Errico et al., 1987). Similarly, a fragment of a transparent rock crystal was recovered in Gudenushöhle, Austria, from an Acheulian layer (Bednarik, 1992). Other quartz crystals in various strata, dating between 276,000 and 500,000 years old, have also been identified in Wonderwerk cave, South Africa (Bednarik, 1993). More recently, Wilkins et al. (2021) reported that early modern humans inhabitants of inland areas collected crystals of calcite 105,000 years ago. Notably, none of those crystals was used as a tool, weapon or ornament. They were neither worked, modified or repurposed in any way, nor do they show signs of use as jewels. Yet, hominins valued these stones enough to transport them from the geological outcrops to the caves used as refuges. These findings suggest that, almost 800,000 years ago— possibly earlier if additional claims are confirmed—, *H. erectus* displayed attraction to quartz and calcite crystals, treasuring them for reasons beyond practical necessity (Bednarik, 2008; Bednarik, personal communication), a behavior that has been regarded as an expression of symbolic thought in populations of *Homo sapiens* (Wadley, 1989; Lewis-Williams and Pearce, 2004; Wilkins et al., 2021). Noticeably, all these crystals collected by hominins were euhedral crystals, i.e., well-formed crystals, with sharp, easily recognized crystal faces. The absence of comparable findings in earlier sites may simply reflect recovery and reporting biases, as small non-utilitarian crystals are rarely detected or recorded in excavations traditionally focused on tools and faunal remains.

This study takes an experimental approach to explore some fundamental questions: Why were hominins drawn to quartz and calcite crystals? Why did they collect them? What properties of crystalline stones attracted them? Designing such a study is challenging, since it is, of course, impossible to conduct experiments with our ancestors. To address this limitation, we adopted a comparative approach (Smith et al., 2018) performing experiments with chimpanzees *(Pan troglodytes)*, one of our two closest living relatives, from which we diverged about six to seven million years ago and with whom we share substantial genetic and behavioral similarities (Takahata and Satta, 1997; Siepel, 2009; Garcia-Ruiz, 2018; Yoo et al., 2025). This methodology is well-established for making inferences about hominin cognitive evolution (MacLean et al., 2012), being fruitful in understanding the roots of language (Erbaba et al., 2025). We sought to determine whether chimpanzees could also exhibit what we have tagged as the “crystal allure”—an attraction to the unique properties of crystals (Garcia-Ruiz, 2018). Our research involved a series of experiments performed in two groups of rescued chimpanzees, living in a semi-captive environment within an ape reserve. We aimed to investigate which characteristics of crystals and rocks could attract the attention of the chimpanzees.

## Methods

### Subjects and facilities

**Figure 1:**
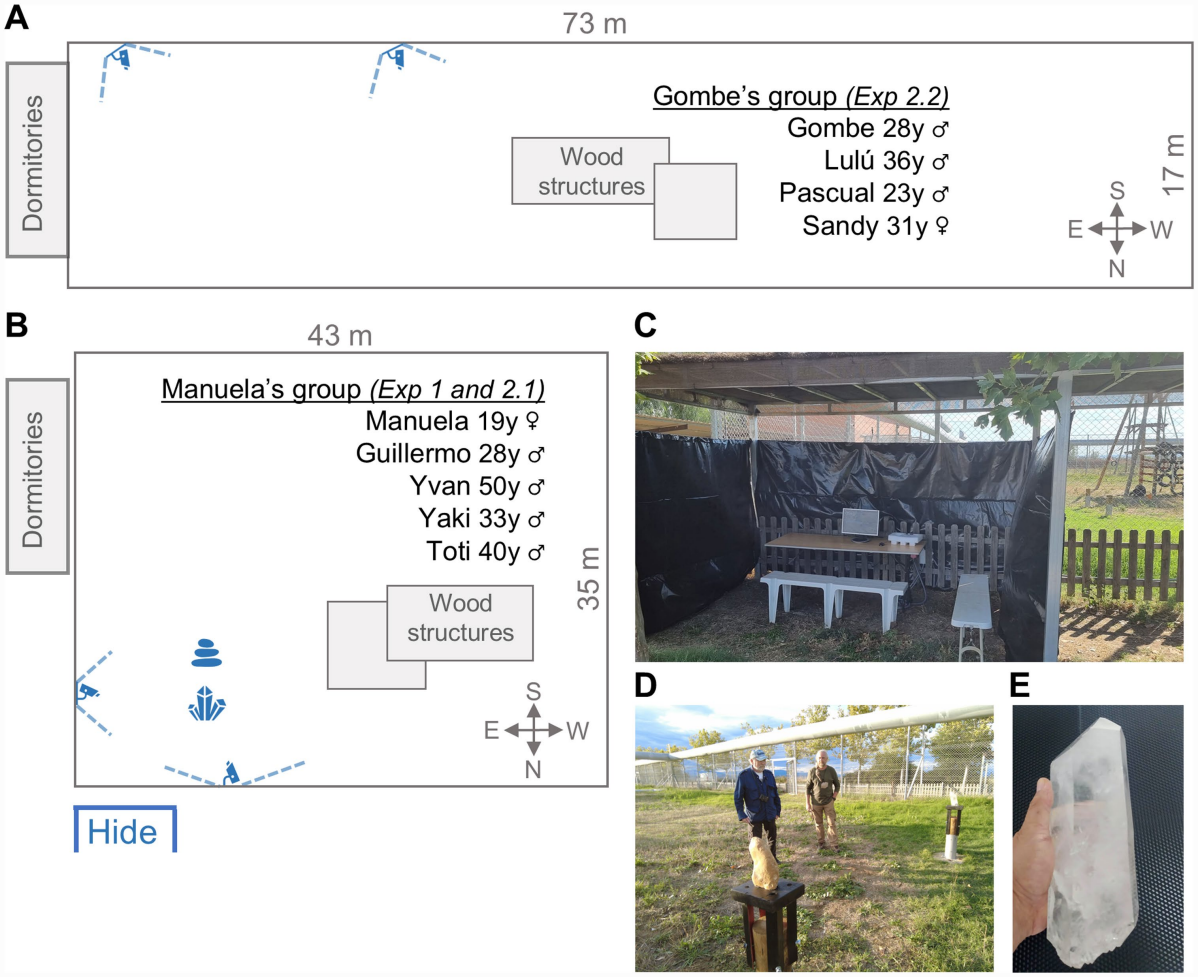
Groups composition, enclosure description, and experimental setup for Experiment 1. **(A)** Sketch of the chimpanzee’s enclosure for Gombe’s group, showing the location of the wood structures, the cameras, and the hide. **(B)** Sketch of the chimpanzee’s enclosure for Manuela’s group, showing the location of the wood structures, the crystal, and the rock used in Experiment 1, the location of the cameras, and the hide. **(C)** The hide with the camera control system. **(D)** The rock and the crystal in their pedestals ready for the experiment; the crystal is on the pedestal in the back and the rock in the pedestal on the front. The authors appear in the background. **(E)** Transparent large single crystal used in Experiment 1. Figure contains images of the author(s) only.

The chimpanzees (*Pan troglodytes*) involved in the experiments were nine adult individuals divided into two social groups (Figures 1A,B; see Supplementary Table S1 for individual profiles). Group 1, referred to as *Manuela*, consisted of 4 males and 1 female (mean age = 34 years; SD = 11.77; range = 19–40) and Group 2, referred to as *Gombe*, included 2 males and 2 females (mean age = 29.5 years; SD = 5.45; range = 23–31).

The chimpanzees were housed in semi-natural indoor and outdoor enclosures with regular feedings schedules. Manuela’s enclosure consists of indoor dormitories (128 m^2^, 6 m high) and an outdoor area of 1,500 m^2^ (Figure 1B). Gombe’s enclosure includes indoor dormitories (64 m^2^, 7 m high) and an outdoor area of 1,300 m^2^ (Figure 1A). Both enclosures were located approximately 20 m apart, and two fences prevented the chimpanzees in one group from seeing or interacting with those in the other group.

The research was primarily conducted outdoors, using surveillance cameras and visual observation from a hide (Figure 1C), with occasional filming and visual observation inside the dormitories. The outdoor grass area features wooden structures, rope installations, rubber wheels, and other enrichment toys. No medical, toxicological or neurobiological research was conducted during the study.

We perform the experiments without altering the everyday routine used by the caretakers of the chimpanzees. Food was distributed twice daily at 11.45 a.m. and 5.00 p.m.; water was available *ad libitum*. Subjects voluntarily participated in the study and were never food- or water-deprived. They did not receive any kind of stimulus during the experimental run. The experiments were performed from September 24 to October 04, 2021.

#### Ethical note

The research was approved by an internal joint ethics committee of the Spanish National Research Council (CSIC—Consejo Superior de Investigaciones Científicas) and was performed in accordance with the recommendations of the Weatherall report “The use of nonhuman primates in research” (Weatherall, 2006).

### Design of the experiments

#### Experiment 1: the monolith

Experiment 1, referred to as “The Monolith,” was designed to investigate the behavior of the enculturated chimpanzees in response to the appearance of two relatively big objects in its living enclosure: a transparent quartz crystal (Figure 1E) and a rock of similar size (Figure 1D).

The objects were located on identical pedestals (Figure 1D), which had been installed months prior to the experiments, so they did not constitute a novelty for the chimpanzees. The setup was designed to ensure that the chimpanzees could not easily destroy the bases but could detach the objects after considerable effort. Thus, the crystal and rock were placed on their respective wooden bases carved with a hole. The lower part of each object was fixed into the hole with polyurethane glue. The wooden base, with either the crystal or rock, was then screwed onto a metal base, which was anchored to a cylinder embedded in a 5 cm deep concrete foundation.

**Figure 2:**
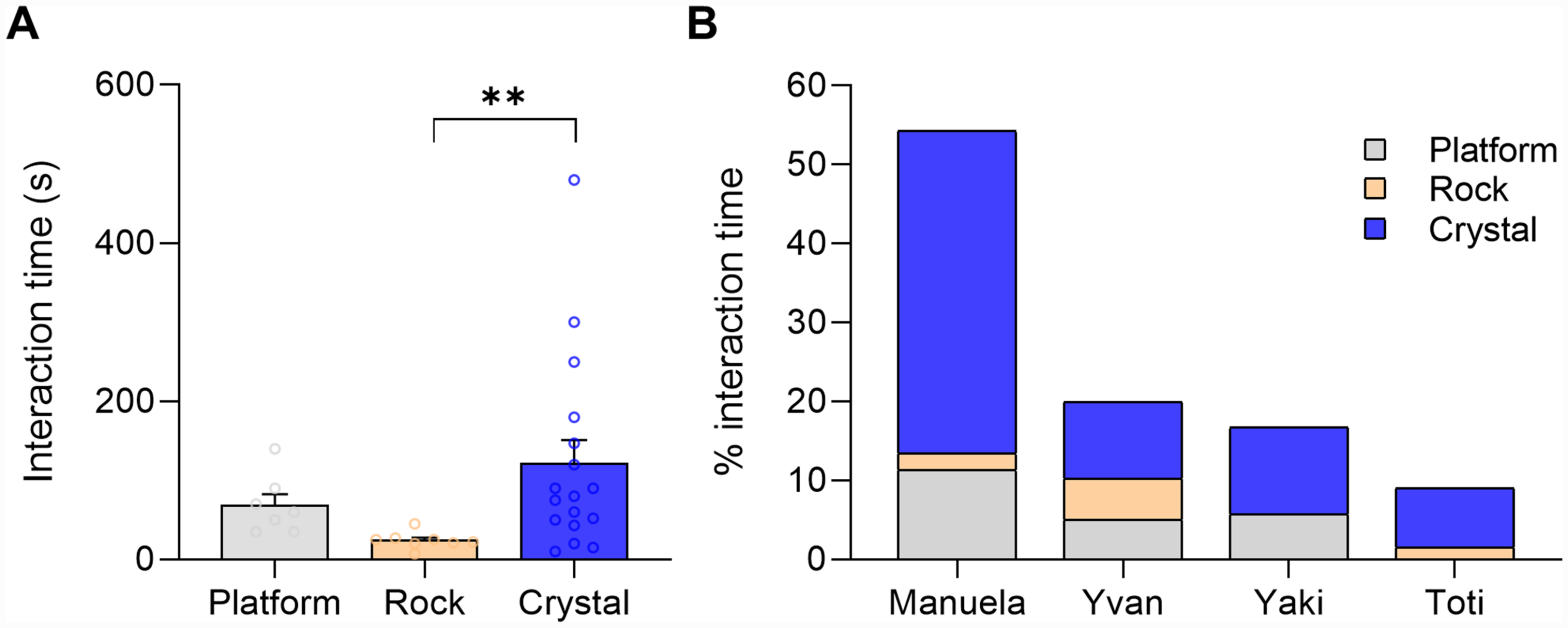
Time of interaction with the crystal in Experiment 1. **(A)** Average interaction time with platform, rock, and crystal (*p* = 0.0052, Kruskal–Wallis test). **(B)** Percentage of interaction time of each individual with the platform, rock, and crystal. The asterisks indicate the level of statistical significance: *p ≤ 0.05, **p ≤ 0.01, ***p ≤ 0.001.

The experiment was recorded using two cameras from 8:30 a.m. to 6:00 p.m., with additional recording of the dormitories with a GoPro camera. To assess the relative attractiveness of the crystal and the rock, interaction times with both of them were recorded and analyzed. Each individual point in the analysis represents a unique interaction of variable duration with either the crystal or the rock (Figure 2A). We quantified the duration of single interactions, which could be visual, tactile, or both. Visual interactions were coded when the subject was clearly looking directly at, or through, the crystal. Interactions were coded by TR, and any dubiety was resolved with JMGR. When an individual moved out of the camera’s field of view, preventing clear observation, the interaction was not assumed to continue during those blind spots. Likewise, when an individual placed an object within its immediate space without maintaining visual or tactile contact, such behavior was not considered an interaction. Less than 5 s interaction were also not considered. This experiment was performed once, with Manuela’s group.

We also repeated this experiment with Gombe’s group; however, it was not possible to quantify any interaction. Shortly after the experiment began, Sandy, from the Gombe group, picked up both objects and carried them inside the dormitories. In this case, no recordings could be made from the interior of the dormitories due to the experimental conditions and the need to ensure the chimpanzees’ well-being.

#### Experiment 2: shapes

Designed to determine if the chimpanzees (1) can differentiate crystals from other stones; (2) the role of crystal transparency and luster in this differentiation; (3) the relative importance of the crystal shape versus transparency in this process.

##### Experiment 2.1: pebbles vs. quartz

**Figure 3:**
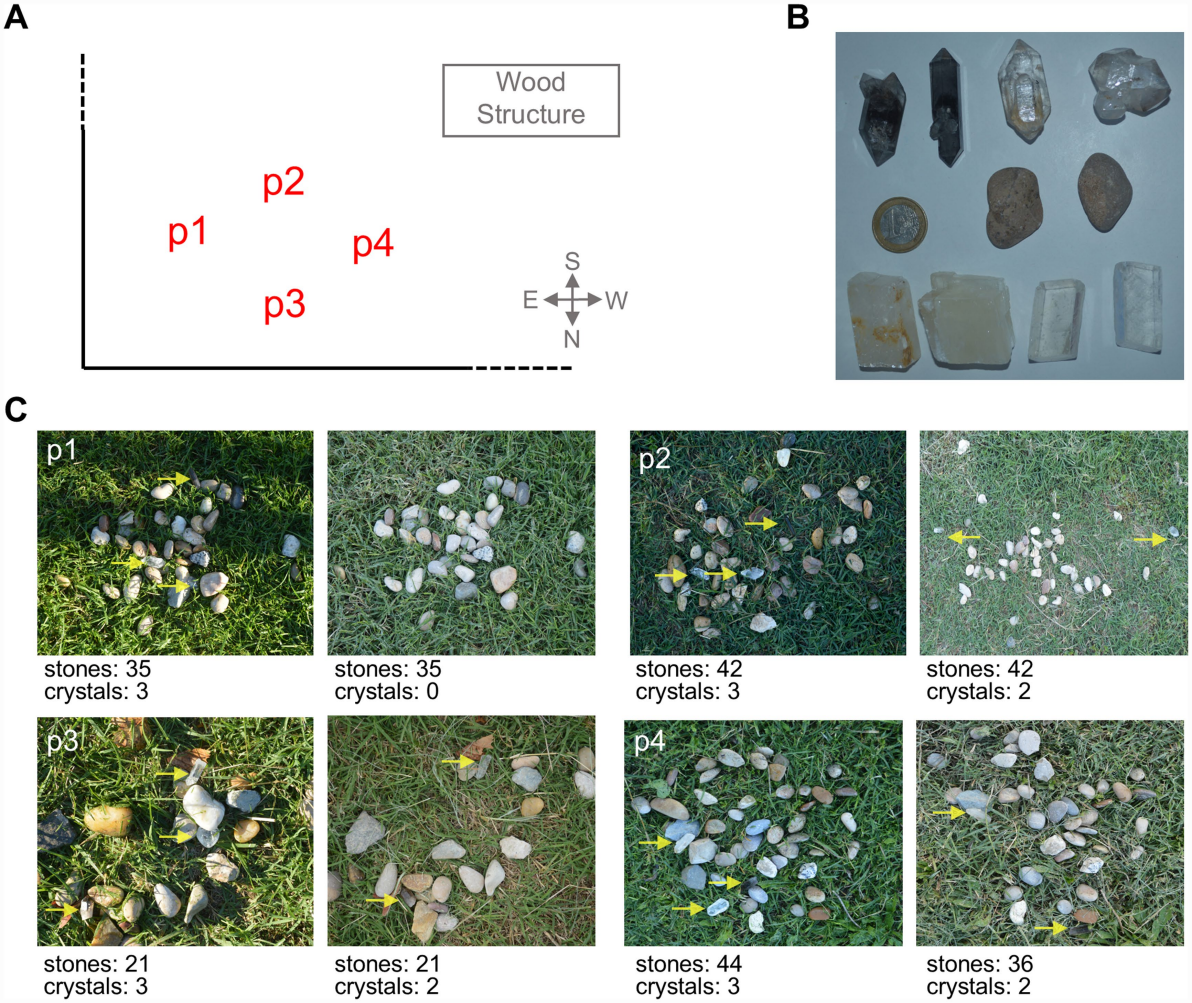
Experimental setup for Experiment 2.1. **(A)** Location of the stone piles in the chimpanzees’ garden of Manuela’s group; **(B)** types of crystals used in Experiment 2.1; **(C)** examples of pebble piles with varying shapes, colors, and textures used in Experiment 2.1. Each pile included one crystal of quartz and one crystal of calcite. Note that we had transparent and translucid crystals of calcite and transparent and opaque (or smoked) crystals of quartz.

We placed four piles of about 20 pebbles of different shapes, colors, and textures on the grass of the chimpanzees’ outdoor enclosure. Each pile contained at least two crystals, including calcite and quartz crystals (Figure 3B; Supplementary Figure S4). Four sets were located in four different locations on the grass near the recording cameras and the observation hide (Figure 3A). This experiment was performed twice with Manuela’s group, using two different sets of crystals in order to explore preferences for transparency and shape (Figure 3B; Supplementary Figure S4).

**Figure 4:**
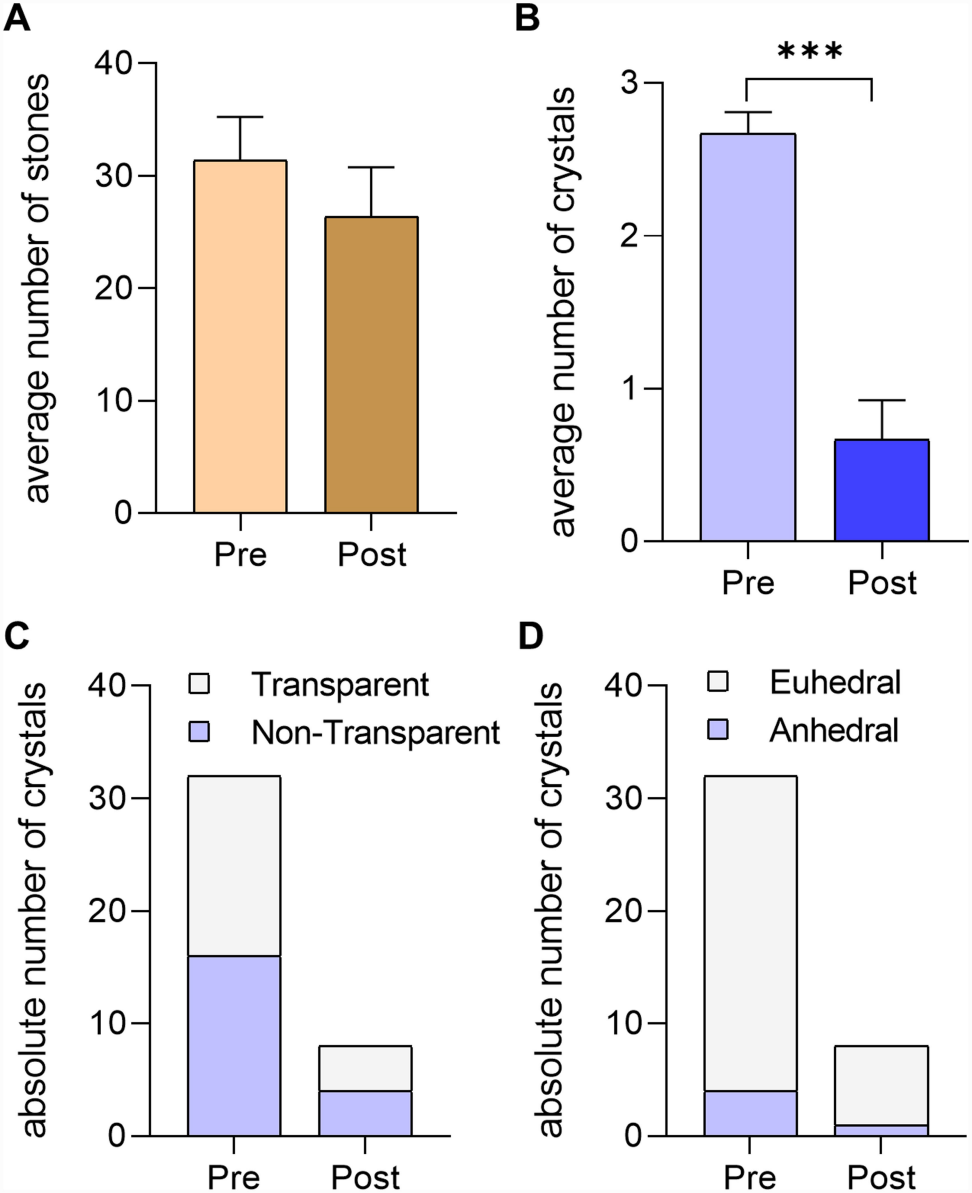
Results of Experiment 2. **(A)** Average number of stones in the piles before and after the experiment. **(B)** Average number of crystals in the piles The Mann–Whitney non-parametric test indicated significant differences (*p* < 0.0001) between the average number of crystals before and after the experiment **(C)** Absolute number of transparent and no-transparent crystals before and after the experiment. **(D)** Absolute number of euhedral crystals before and after the experiment.

We took pictures of the sets of pebbles and crystals before the chimpanzees entered the outdoors and after their manipulation, when they retired to their dorms (Figure 3C). The difference in the number of crystals and pebbles between the images was quantified, and the average number of stones in each pile, as well as the crystal to stone ratio, was calculated. This data was then statistically analyzed and used to interpret the results (Figure 4).

##### Experiment 2.2: pebbles vs. pyrites

**Figure 5:**
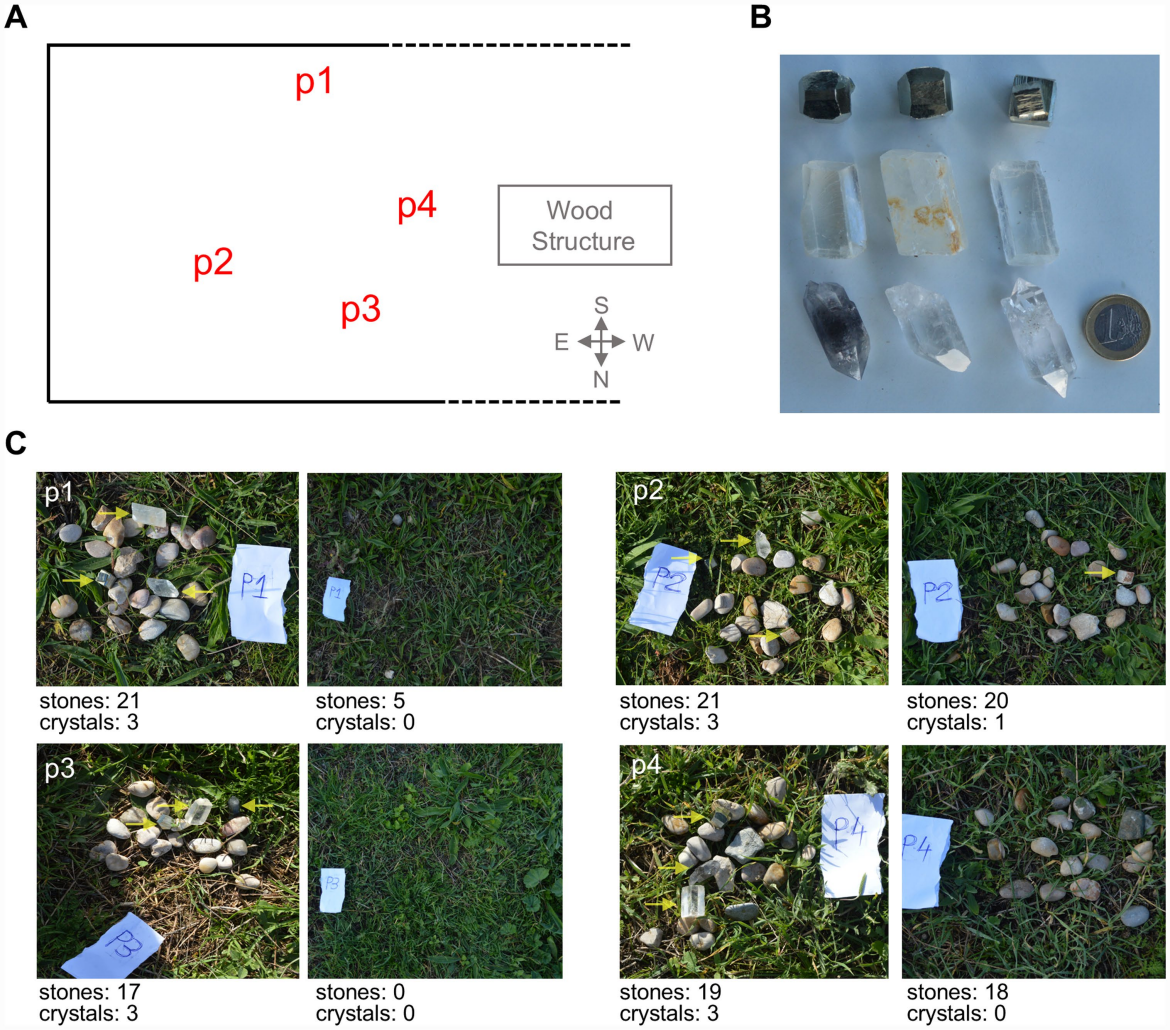
Experimental setup for Experiment 2.2. **(A)** Location of the stone piles in the chimpanzees’ garden of Gombe’s group. **(B)** Types of crystals used in Experiment 2.2. **(C)** Examples of piles of pebbles with varying shapes, colors, and textures used in Experiment 2.2. Each pile included one crystal of quartz and one crystal of pyrite.

We placed four piles of about 20 pebbles of different shapes, colors, and textures on the grass of the chimpanzees’ outdoor enclosure. Each pile contained at least two crystals, including quartz and pyrite crystals (Figure 5B). Four sets were located in four different locations on the grass near the recording cameras and the observation hide (Figure 5A). This experiment was performed with the Gombe group.

We took pictures of the sets of pebbles and crystals before the chimpanzees entered the outdoors and after their manipulation, when they retired to their dorms. For the subsequent statistical analysis, the total number of stones and crystals in each pile was counted before and after the experiment (data shown in Figures 3C, 5C). The initial number of crystals in each pile was always three, whereas the number of stones varied around thirty-five. The average values of the four piles are represented in Figures 4A,B, and the absolute number of crystals is represented in Figures 4C,D.

### Experimental materials

#### Experiment 1: crystals and rock

The large crystal used in Experiment 1 is an elongated quartz crystal weighing 3.3 kg and 35 cm in height (Figure 1E). It was collected from Zigras Mine in Blue Springs, Garland County, Arkansas. The crystal displays well-developed faces and edges, it was transparent with no evidence of fluid inclusions to the naked eye. The crystal morphology was dominated by the {10-10} prismatic faces of the hexagonal prism topped with the {2-1-11} faces of the left-handed hexagonal bipyramid. Only one of the ends of the crystal shows well-developed bipyramidal faces. The stone used in Experiment 1 was a brownish sandstone of elongated shape with continuous curvature with a weight of 2.1 kg and 34 cm in height (Figure 1D).

#### Experiment 2: shapes

##### Experiment 2.1: crystals and pebbles

We used six types of crystals in Experiment 2.1 (Figure 3B; Supplementary Figure S4):

(a) Clear euhedral quartz crystals (Figure 3B, upper row right)
(b) Opaque euhedral quartz crystals (Figure 3B, upper row left)
(c) Transparent calcite crystals showing birefringence (Figure 3B, bottom row left)
(d) Milky calcite crystals (Figure 3B, bottom row right)
(e) Anhedral (rounded) transparent quartz crystals (ESM, Supplementary Figure S4)
(f) Pebbles (Figure 3B, middle row)

Calcite and quartz crystals were selected for their similarity in size and crystal quality to those collected by hominins tens to hundreds of thousands of years ago. Both types were in the range of 2.5–3.5 cm. Calcite crystals were rhombohedric obtained by cleavage, while quartz crystals were not ended by bipyramid, but by a single pyramid at one of its ends.

##### Experiment 2.2: quartz and pyrite crystals

We used three types of crystals in Experiment 2.2 (Figure 5B):

(1) Pyrite crystals, cubic system, cube morphologies, fully opaque with a metallic luster (Figure 5B, top row). We selected crystals of pyrite from the locality of Ambasaguas (Spain) because they are euhedral single or twined crystals with clear cube (hexahedra) shape. Measures were in the range of 2–3 cm.
(2) Calcite crystals of rhombohedric shapes have two different levels of transparency (Figure 5B, middle row).
(3) Quartz crystals of hexagonal symmetry, with prismatic and bipyramidal shapes, and a vitreous luster (Figure 5B, bottom row).

### Recording images and videos

Four video cameras were installed near the area used for the experiments (Figures 1A,B). Two of them were fixed outdoor digital cameras AirSpace PRO, and two digital cameras 2mp 1080p outdoor motorized PTZ HD-CVi. The cameras were coupled to a Pentahybrid VCR HD-CVi and IP with four analogical channels. The cameras were located on the railing at four meters high. Two of the cameras were not orientable and only could zoom. The other two cameras can be oriented and zoomed. The four cameras were driven by a computer and were handled by the team from inside a hide built two meters far from the railing closest to the experimental area (Figure 1E). Under some circumstances, when the chimpanzees remained inside the dormitories, a GoPro camera was cautiously placed to record their behavior. All the experiments add up to a total of 72 h of footage.

### Statistical analyses

The data were analyzed using GraphPad Prism^®^ 9.0 software. The normality assumption was checked using the Shapiro–Wilk test. The unit of analysis were the duration (s) of individual interactions with the platform, rock or crystal from Experiment 1, and the number of stones and crystals removed from each pile from Experiment 2. None of these data followed a normal distribution, so non-parametric tests, like Mann–Whitney or Kruskal-Wallis, were performed to compare differences between means. Data in graphs are presented as Mean + SD. Statistical significance threshold was taken as *α* = 0.05.

## Results

### Experiment 1-the monolith: preference for crystal vs. rock

In this experiment, the subjects were presented with a transparent quartz crystal (Figure 1E) and a shapeless rock with curved contours, each placed on top of two pedestals, about 70 cm high (Figure 1D).

Initially, both objects along with pedestals caught the attention of the chimpanzees. However, as illustrated in Figure 2, the chimpanzees displayed a preference (*p* = 0.0052, *H* = −12.59, Kruskal–Wallis test) for interacting with the crystal. After several attempts, the chimpanzees succeeded in removing the crystal and the rock from their pedestals. Yvan, from Manuela’s group, pulled out the rock. Neither he nor any other individual, including the alpha female, devoted more than 2 min of attention to the large rock. Once removed, the rock was abandoned on the ground for 38 min before being transported ca. 10 m from its original location by Yvan, where it remained untouched for the rest of the experiment. In the case of the large crystal, all individuals (*N* = 5) from the Manuela group interacted with the crystal while it was on the pedestal, later in the wooden platform, and in the dorms. After several attempts by various group members, Manuela, the alpha female, and the strongest chimpanzee, successfully removed the crystal from the pedestal. She then left the crystal on the ground. Immediately, Guillermo approached Manuela slowly, looking at the crystal, but then Manuela took it and carried the crystal to the top second floor of the wooden platform. After briefly playing with the crystal, she moved to the third floor but left the crystal on the second floor. Instantly, Yvan (and soon later, Yaki) appeared, carefully handling and examining the crystal. Eventually, Yvan took the crystal and transported it to the dorm (see Supplementary Video S1), where they keep it for a couple of days until we managed to retrieve it.

**Figure 6:**
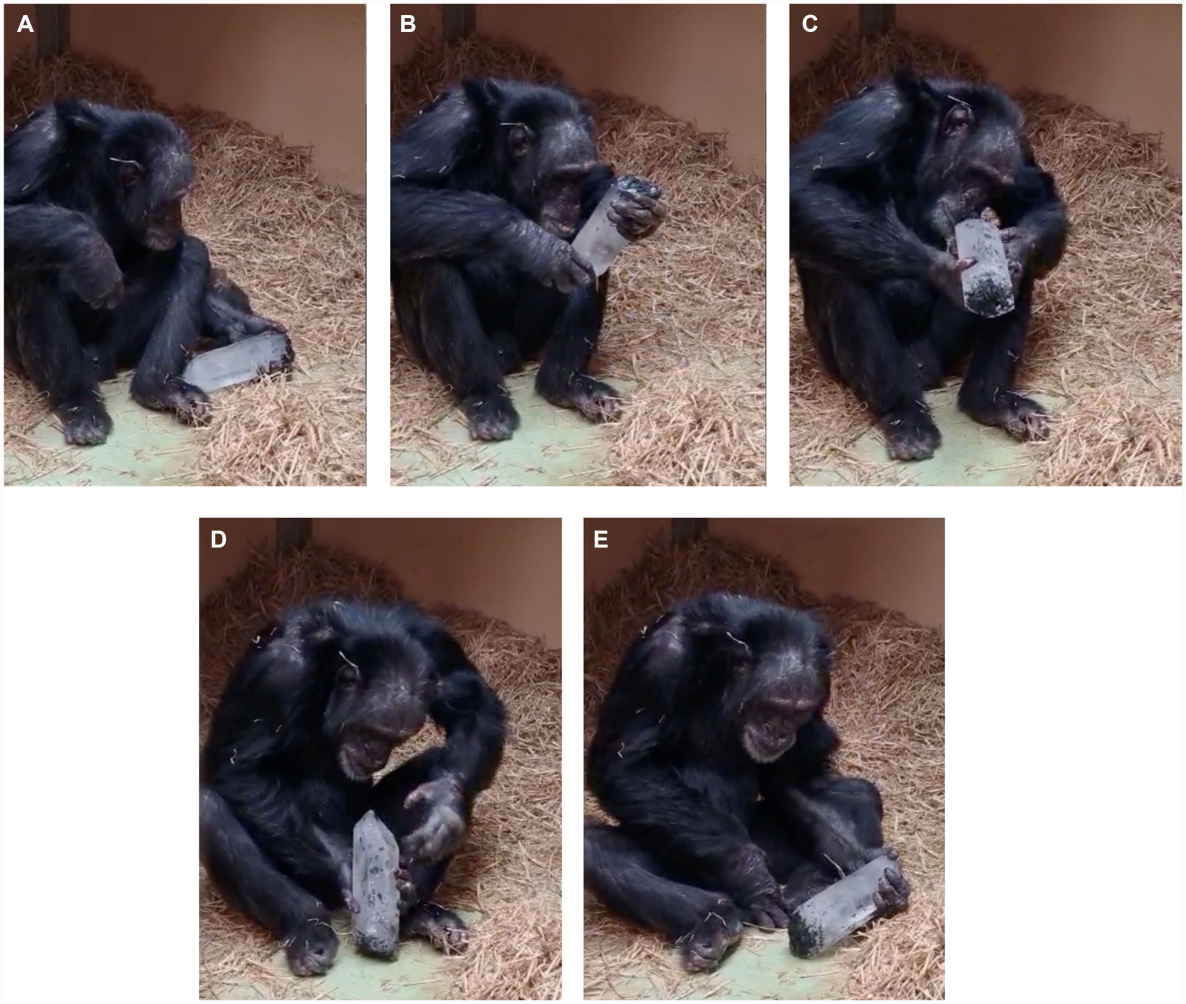
Frames of Toti’s attentive observation of the quartz crystal. **(A–E)** Frames from the footage showing Toti’s interaction with the quartz crystal during Experiment 1.

We video-recorded most of the chimpanzees’ activities in the dorm while the crystal was present. Our records show that Manuela, Yvan, Toti, and Yaki from the Manuela’s group, handled and inspected the crystal. In Figure 6, we selected frames from Supplementary Video S2 showing Toti attentively observing the quartz crystal. She rotates it in her hands and tilts her head to view the crystal from specific angles, displaying particular interest in the view aligned to the hexagonal axis.

The total time spent manipulating the crystal was significantly higher than that from the rock (*p* = 0.0052, *H* = −12.59, Kruskal–Wallis test) (Figure 2A). All four individuals engaged in manipulation proportionally more with the crystal compared to the rock or the platform (Figure 2B).

We also observed Sandy hitting the pyramidal tip of the crystal against another crystal (Supplementary Video S3), doing so delicately and without any apparent intention of breaking it. This behavior was also observed, though not recorded, in Toti. Supplementary Figure S1 shows the crystals after a day in the hands of chimpanzees in their dormitory. It only shows a small break in the tip after hitting the floor with the crystal. The fact that the chimpanzees we tested preserved the large crystal in their enclosure for 2 days suggests the object was valued. Furthermore, when the team of caretakers tried to retrieve the crystal, it took hours to exchange it for valuable “gifts” (i.e., favored food items—bananas and yogurt—which are known from daily observations to be highly appreciated by the chimpanzees), which also suggests that the crystal was highly valued.

### Experiment 2-shapes

#### Separating crystals from pebbles

The results of Experiment 2 revealed that the chimpanzees we tested are capable of identifying and selecting individual euhedral crystals of calcite and quartz from among pebbles of different shapes and lithologies (*p* < 0.0001, *U* = 6.00, Mann–Whitney test) (Figure 4B). Supplementary Video S4 shows Guillermo selecting crystals from the pile with the mouth, completing the task within seconds. The chimpanzees exclusively selected the quartz and calcite crystals, leaving the other pebbles in the grass (Figure 3C).

**Figure 7:**
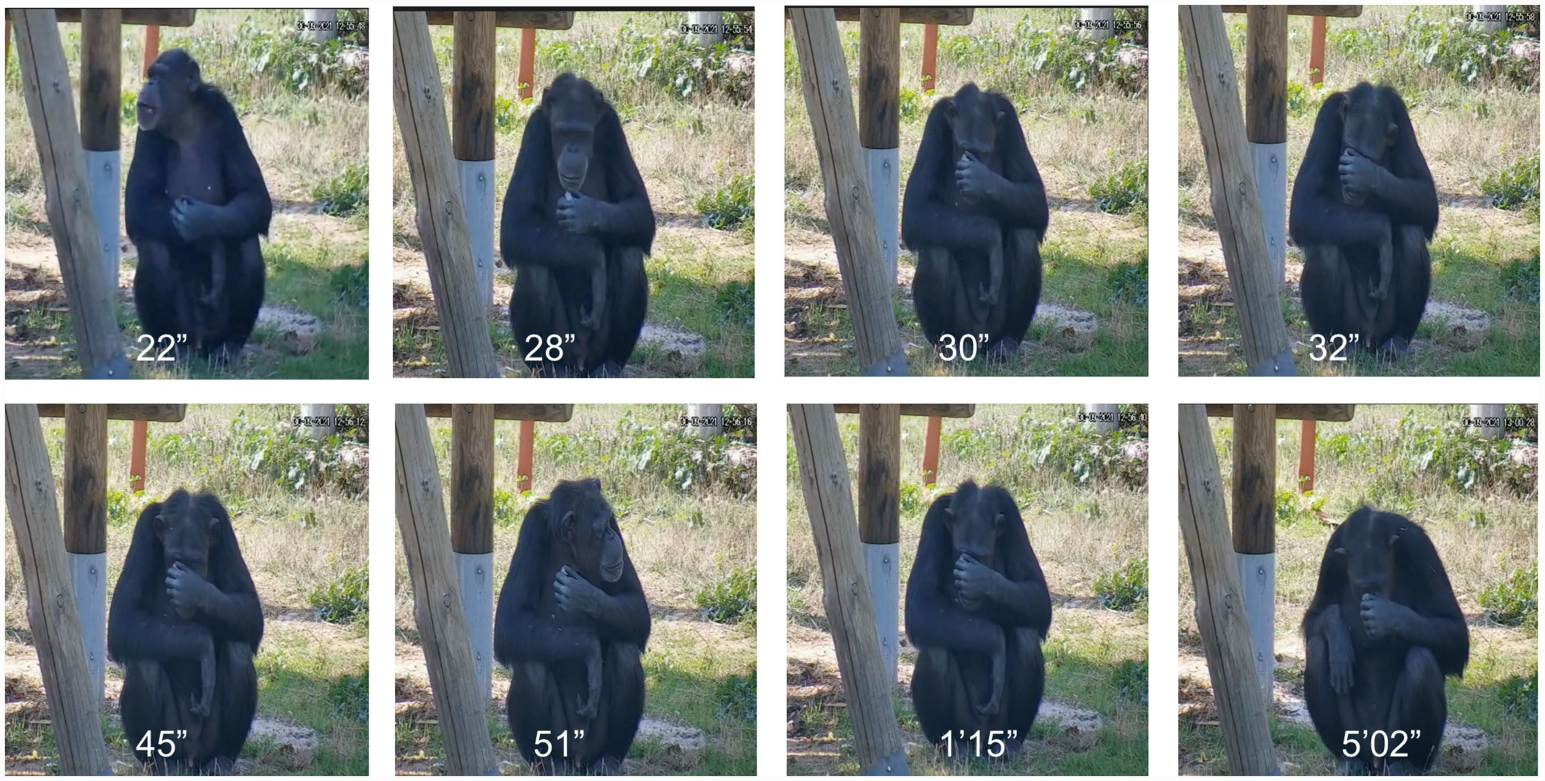
Frames of Yvan interaction with small crystals. In this series of frames (Supplementary Video S5), we see Yvan, who picked one of the crystals of quartz that we deliberately located in their garden within a pile of pebbles. Yvan has a transparent crystal of quartz of ca. 3.5 cm in size in his left hand. He then brought the crystal very close to his eye and inspected it carefully. The numbers in the pictures indicate the time frame in the video. He repeated the actions several times. This episodic inspection lasted for more than 15 min. Yvan also did the same inspection of transparency inside the dorm (Supplementary Figure S3; Supplementary Video S6).

We could not determine the ultimate fate of the selected crystals, as tracking their locations without disturbing the chimpanzees and their caretakers’ routines was not feasible. However, observations from Experiment 2 suggest that the chimpanzees’ interest in crystals goes beyond novelty. In Figure 7 (frames from Supplementary Video S5), Yvan is shown inspecting a transparent quartz crystal of ~ 3.5 cm, retrieved from a pile of pebbles in the outside facility. He held the crystal close to his eye and examined it intently for over 15 min, with repeated observations averaging 35 s, the longest lasting 1 min. Since Yvan is not myopic, this behavior suggests a focused and genuine interest, particularly in crystal transparency.

This behavior was repeated by Yvan both outside (Figure 7; Supplementary Video S5) and inside the dorms (Supplementary Figure S3; Supplementary Video S6). Toti, when selecting crystals from the piles, also inspected them close to his eye, similar to Yvan (Supplementary Video S7). The chimpanzees continued inspecting the crystals while relaxing inside the dorms (Supplementary Figure S2) and also positioned themselves near the window to observe the crystals against the light (Supplementary Figure S3). A detailed analysis of the videos and our direct observations confirmed that the crystals being examined were quartz. While the chimpanzees were observed holding the crystals from a distance of about half a meter, they also held the crystals close to their eyes, suggesting some interest in their optical properties in addition than the reflections on the crystal faces.

#### Transparency versus morphology

To further investigate the role of transparency versus morphology, we performed Experiment 2.2, which involved adding pyrite crystals to the piles of pebbles in addition to the quartz and calcite crystals. Pyrite crystals have a cubic shape, different from the trigonal and rhombohedral shape of quartz and calcite crystals (Figure 5B). Most pyrite crystals appear in nature as crystal aggregate.

Regarding optical properties, pyrite crystals have a metallic luster different from the vitreous luster of quartz and calcite. Furthermore, pyrite crystals are opaque in contrast with the transparency of quartz and calcite.

**Figure 8:**
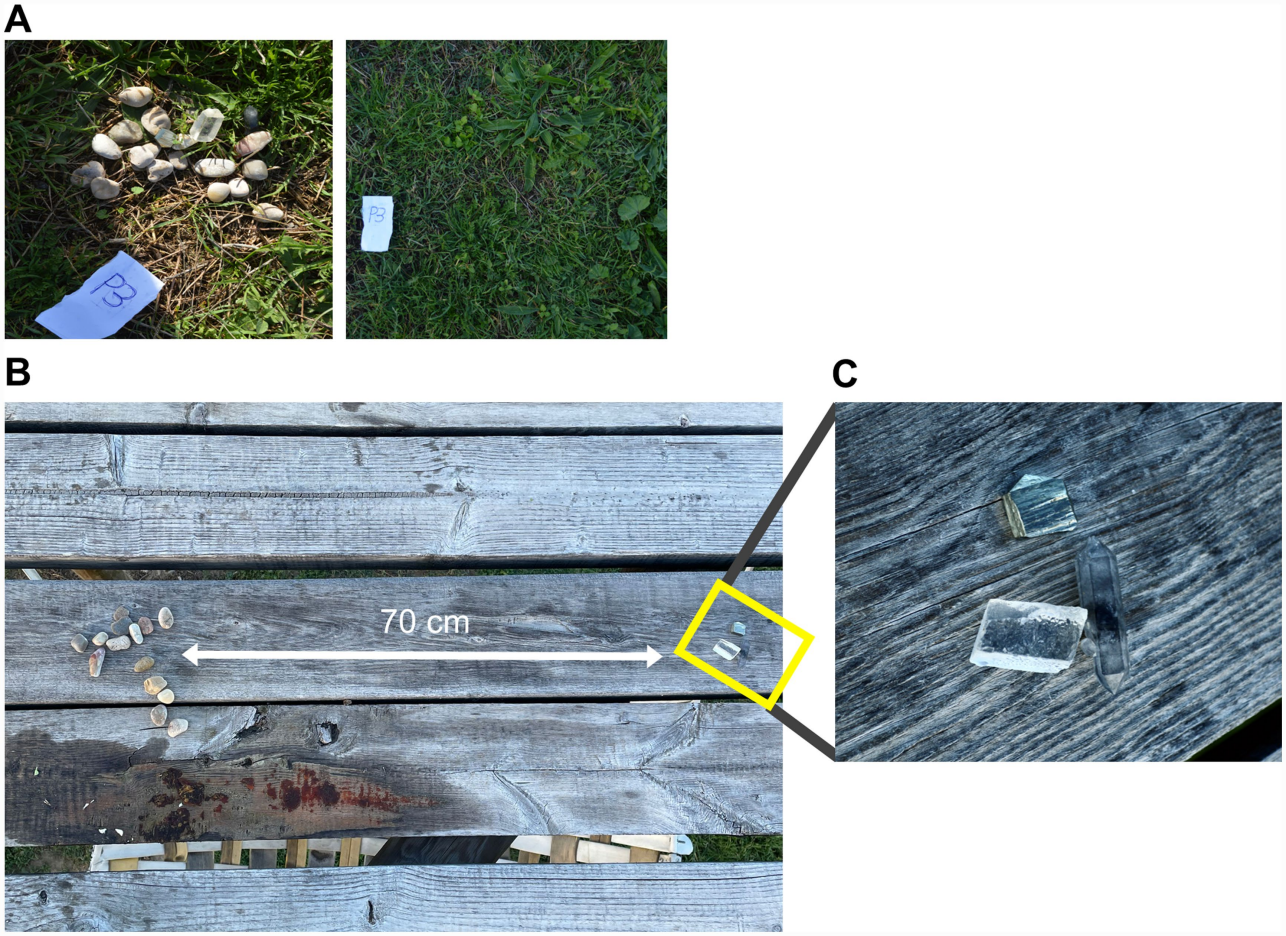
Crystal discrimination performed by Sandy in Experiment 2. **(A)** The original pile of pebbles and crystals; **(B)** Sandy’s separation of the three crystals from the pile of pebbles; **(C)** close-up view of the three separated crystals: quartz (right), pyrite (up), and calcite (bottom left).

Experiment 2.2 yielded another interesting result. As shown in Figure 8A, in pile P3 all items disappeared after the chimpanzees interacted with the pile. Everything, pebbles and the three crystals. Video recording shows that one of the chimpanzees, Sandy, from the Gombe group, first picked crystals from P3 and P4, placing them in her mouth. She took them to the middle level of the wooden structure, where she manipulated the stone and crystals she had collected. Eventually, Sandy returned to piles P3 and P4 for more pieces, this time carefully analyzing each crystal or pebble before deciding whether to pick it or not. Upon finishing, she returned to the wooden structure carrying both crystals and stones and remained there manipulating them. For the rest of the day, she was observed carrying crystals in her mouth. When we searched for the results of Sandy’s manipulation, we discovered that she separated the ensemble into two groups of pieces far from each other: one group consisting of pebbles and the three crystals of calcite, quartz, and pyrite in another (see Figure 8).

In summary, Experiment 2 yielded interesting results. Experiments 2.1 and 2.2 showed a significant decrease in the number of crystals in the piles after the experiment (Figure 4B). Correspondingly, the number of non-crystalline pebbles in the piles before and after the experiment did not exhibit significant differences (Figure 4A). In the Experiment 2.1, when we placed transparent and non-transparent crystals among the pebbles, the chimpanzees identified and collected the same percentage of both types of crystals (Figure 4C). In the Experiment 2.2, when we included euhedral transparent quartz crystals along with anhedral transparent quartz crystals (polished crystals where the crystal faces are not identifiable), we observed that the chimpanzees took both (Figure 4D).

## Discussion

In this exploratory study, we investigated whether two groups of enculturated chimpanzees show attraction to crystals and which specific properties may influence this interest. Our motivation stems from the longstanding archaeological evidence that hominins collected crystals for at least 780,000 years. We wondered whether some of the perceptual or cognitive tendencies that make crystals appealing to humans might also be present in other hominins. To explore this, we conducted a series of group-based experiments with chimpanzees (*Pan troglodytes*) in a sanctuary setting.

In Experiment 1, the interest aroused by the crystal was much greater than that of the rock and pedestals. As Figure 2 shows, the interaction time with the crystal is much longer than with the rock and pedestal in the outdoor garden. Furthermore, the chimpanzees took the crystal into the dormitories, ignoring the rock, and actively interacted with the crystal for almost 2 days. Supplementary Video S2 shows one of the chimpanzees (Toti) sitting on the floor, rotating the large crystal in his hands and tilting his head to look at the crystal from specific orientations.

In Experiment 2, the individuals quickly and consistently chose crystals, even when their transparency, luster, and shape varied. In those cases where we were able to measure it, the identification and selection of the crystals took only a few seconds, including the first experiments, when the chimpanzees saw the piles of crystals and pebbles for the first time.

Identifying the property that allows chimpanzees to distinguish crystals from pebbles is not straightforward. In our piles of pebbles and crystals, we placed transparent and translucent quartz and calcite crystals. In Experiment 2.1, when we placed transparent and non-transparent quartz crystals among the pebbles, the chimpanzees identified and collected both types (Figure 4C), suggesting that shape is a key attractive property for them. In another experiment (included in Experiment 2.1), we included three crystals in each pile: euhedral transparent quartz, anhedral transparent quartz, and calcite translucent crystals. We observed that the chimpanzees took both anhedral and euhedral crystals (Figure 4D), suggesting that transparency might be more attractive. In combination, the results of these two experiments suggest that transparency and shape are both attractors for crystal allure.

In Experiment 2.2, we included in the piles of pebbles three crystals of different symmetry, luster, and transparency: cubic pyrite crystals with metallic luster, rhombohedral milky calcite crystals with pearly luster, and transparent hexagonal quartz crystals with vitreous luster. Of the 12 crystals included in the four piles, they picked up 11, regardless of luster, shape, or transparency. The separation of pebbles and rock that Sandy performed in the platform suggests that she recognized the geometric similarity among the three crystals and differentiated them from the rounded pebbles (Figure 8).

The importance of transparency is dramatically demonstrated by analyzing the behavior of chimpanzees with quartz crystals collected from the piles. Supplementary Videos S5, S6 shows Yvan’s specific interest in crystal transparency, both inside and outside the dormitories. Behavior also observed in Toti (Supplementary Video S7). We confirmed with the Rainfer staff that Yvan had no vision problems, so we hypothesize that this prolonged examination demonstrates that crystals evoke an exploratory interest in Yvan.

Taken together, these results suggest that transparency and morphology act as perceptual attractors. Although our data do not allow us to determine the relative contribution of each property (i.e., transparency and morphology) in all individuals, the experiments show that both features can guide object selection. Both characteristics are uncommon in natural objects, which may facilitate rapid detection and trigger attraction. Transparency played a significant role in capturing hominin attention, as it does today with humans (Worringer, 1997; Scheerbart, 1998; Garcia-Ruiz, 2018). Hominins were familiar with transparency probably because water, under certain circumstances, is transparent. The etymology of the word “crystal,” which means ultra-frozen water (Amorós and Tavira, 1983), highlights the relevance of optical properties in the conceptual connection humans made between transparent minerals and water. Thus, the combination of transparency and solidity in quartz crystals likely made them especially captivating, as these were the only solid objects encountered by early hominins that exhibited striking optical characteristics, such as high transparency and light reflection.

The collection of non-transparent crystals by hominins 105,000 years ago in the Kalahari Desert (Namibia) (Wilkins et al., 2021) suggests that transparency alone was not the sole property that attracted hominins. This can now be explained because our results suggest that the morphological singularity of crystals likely played a role in their appeal. When *H. erectus* tried to make sense of their environment, their cognitive processes were naturally drawn to patterns, separating what was similar from what was different (Shultz et al., 2012). The natural world in the savannah was dominated by curves and branching structures of fractal geometry (Mandelbrot, 1982): trees, bushes, water-carved furrows, streams, clouds, mountains, animals, and even their co-specifics. There are very few natural objects with straight lines and flat surfaces. Indeed, there are no polyhedral objects, except crystals. When *H. erectus* discovered quartz and calcite crystals, they encountered euhedral objects, which are markedly different from all other objects and from the organic norm of their surroundings. Crystals, strange pebbles with straight lines, flat faces, and precise angles, free of curves, were unique in appearance. Except for crystals, straight lines, euhedral, and objects with Euclidean geometry were all invented by humans.

The discovery of crystals may have sparked early cognitive recognition of forms and patterns beyond the immediate natural world, underscoring their appeal. It may have facilitated the early perception of symmetry and the production of symmetrical artifacts. This development is part of the broader evolution of hominid spatial perception-cognition, which includes both developments in perceptual-cognitive abilities and developments in motor skills (Wynn, 2000; Coolidge, 2020). Archeological studies on hominin tool production (Malafouris, 2021) indicate that the cognitive requirements of stone knapping are within the abilities of apes at a very basic level, yet they seem unable to do so without human help (Motes-Rodrigo et al., 2022). The earliest artifacts produced by hominins—the Mode I tool industry, dating to 2.5 million years ago—do not exhibit clear symmetrical patterns. The earliest examples of hominid-imposed symmetry appear on hand axes, which date back 1.4 million years, and there was a significant improvement in symmetry around 500,000 years ago, coinciding with the time when hominins began collecting crystals (Machin et al., 2007).

### Future research

Future research should test wild, unenculturated apes, as well as other great apes species, especially bonobos and gorillas, to assess the extent of crystal-directed interest across the Hominidae. Comparative studies could clarify whether “crystal allure” reflects a shared perceptual bias or arises under specific ecological or developmental conditions. Field studies in earlier *Homo* archaeological sites and future experiments with other ape species will shed light on how ancient crystal allure really is. Combining behavioral experiments with archaeological and cognitive evidence may ultimately elucidate whether certain physical properties of crystals influenced early perceptual or conceptual tendencies in hominins. Behavioral studies on the preferences of hominins and non-hominin apes for symmetrical patterns have never considered the attraction that crystals exerted on hominins. Incorporating insights from experiments on crystal allure is meaningful but requires caution.

Furthermore, our work raises new questions within the framework of the research on the impact of the material world on the evolution of the mind (Criado-Boado et al., 2019; Malafouris, 2021). According to this novel approach, crystals being singular natural objects recognized and valued by hominins for at least hundreds of thousands of years may have influenced our comprehension of the natural world. Our results could help explain preferences for regular visual patterns and symmetrical stimuli without specific training (Rensch, 1973; Anderson et al., 2005). To what extent have crystals become cognitive extensions of the human body? Do we humans exhibit a preference for order because the Euclidean geometry of crystals attracted us? Do we pursue ancestral cognitive preferences for patterns, regularity, and order? Were crystals the catalysts for the neuronal processes that led to the ability to abstract and use abstraction for understanding the world? All these are important questions that demand further investigation in this research line.

Abstraction and geometric styles shaped the earliest artistic expressions (Rodríguez-Vidal et al., 2014; Leder et al., 2021), for which crystals may have played a role. This notion aligns with proposals by Hodgson (2006, 2020), who suggested that the structured, repetitive nature of crystals provided cognitive templates for artistic abstraction. Similarly, Worringer and Kramer’s (1997) theories anticipated the connection between humanity’s fascination with geometric forms and the natural world’s underlying order. The crystals found in *Homo* settlements do not appear to have been used ornamentally (since they are not perforated) or as tools or weapons (since they are not carved), but this does not rule out a symbolic use, for ritualization or even with animistic meaning. Therefore, crystals may have contributed to the development of metaphysical and symbolic thinking, acting as catalysts for the conceptualization of a “big beyond.” Their rarity, optical allure, and geometrical singularity could have imbued them with special meaning, serving as physical representations of ideas beyond the immediate and tangible world.

### Limitations

Several limitations of our study must be acknowledged. Our results cannot be fully interpreted statistically, as the sample size was limited to two groups of five and four chimpanzees, and individual cognitive variation due to differences in personality and life history, although not explicitly measured here, was substantial (see Supplementary Table S1). The specific characteristics of the Rainfer center did not allow for an individualized study. Still, it was estimated that a group test should be conducted, as better results have been reported when individuals were tested in a group than when tested individually (Anderson et al., 2005). Despite our initial intention to replicate each experiment across both groups, restrictions on recording and animal interactions prevented full replication. Most of the Rainfer chimpanzees came from circuses, while others were raised among humans. For this reason, we cannot know whether these chimpanzees are representative of wild chimpanzees, although it cannot be ruled out that their responses to the crystals are the same. The Rainfer chimpanzees involved were familiar with Euclidean objects made by humans. They are familiar with objects with straight lines, fixed angles, transparency, and flat, bright surfaces. This prior exposure is a relevant characteristic of our sample. Therefore, our result should be confirmed and extended through future behavioral investigations with wild apes.

## Data Availability

The original contributions presented in the study are included in the article/Supplementary material, further inquiries can be directed to the corresponding author.
