# Supplementary material for: On the origin of our fascination with crystals
Source: Front Psychol. 2026 Mar 4;17:1633599. doi: 10.3389/fpsyg.2026.1633599 (PMC12995665; doi:10.3389/fpsyg.2026.1633599)
Supplement: Supplementary file 1 [file Data_Sheet_1.pdf]

Table S1: *Individual Background Information of Participant Chimpanzees*

Group	Name	Sex	Entry Date	Age (Years)	Description
Manuela	Yvan	M	09.05.2009	50	Protruding mouth, playful, careful, and very sociable. Not aggressive or fearful. Shows reciprocity and comforting behaviors, emotionally dependent on Manuela.
Manuela	Toti	M	16.01.2007	40	Honey-colored eyes, hairless shoulder, arthritis in legs, often crawls on the floor.
Manuela	Yaki	M	11.09.2004	33	Thin with long arms, has arthritis. Very sociable, mildly aggressive, somewhat fearful, and not very curious. Shows prosocial behaviors with humans.
Manuela	Guillermo	M	05.07.2007	28	Blind in one eye, not sociable, somewhat aggressive, very fearful, and avoids contact. Lacks curiosity, sways when nervous.
Manuela	Manuela	F	28.12.2001	19	Alpha female. The only individual born within the Foundation. Physically and mentally healthy, sociable, curious, minimally aggressive. Displays anthropomorphic behaviors and uses some sign language.
Gombe	Lulú	F	10.06.2005	36	Thin, walks cautiously. Very sociable, non-aggressive, not very fearful, and quite curious.
Gombe	Sandy	F	09.12.2007	31	Dark hair, decisive, often shows maternal behavior with objects. Very sociable, highly curious, non-aggressive, and not fearful. Displays anthropomorphic behaviors and protects her objects.
Gombe	Gombe	M	09.12.2007	28	Alpha male with white hair on the back, phantom limb, and periodic neurotic crises. Not sociable, quite aggressive, fearful, and not curious. Reacts to novelties with aggression.
Gombe	Pascual	M	15.04.2008	23	Not very sociable, lives isolated, often eating. Interacts minimally with females, not curious, somewhat aggressive and fearful. Struggles to respect the alpha male's hierarchy, especially regarding food.

Note. This table provides the demographic and background details for all chimpanzees involved in the study. "Group" is designated by the name of the alpha individual. Individuals are listed in descending order of age within their group. "Entry Date" refers to the date the individual entered the care facility (DD.MM.YYYY format). Manuela is the only participant who was born within the facility; all others were integrated at various later dates. "Description" summarizes key health and behavioral observations provided by caretakers.

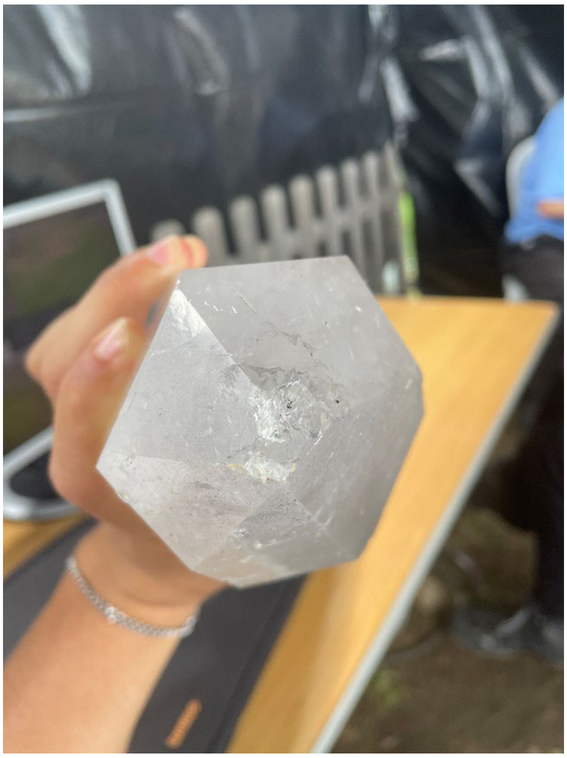

Fig S1. Quartz crystal after being hit by Sandy. Photograph showing the quartz crystal following the impact delivered by Sandy to its tip, as recorded in Video S3.

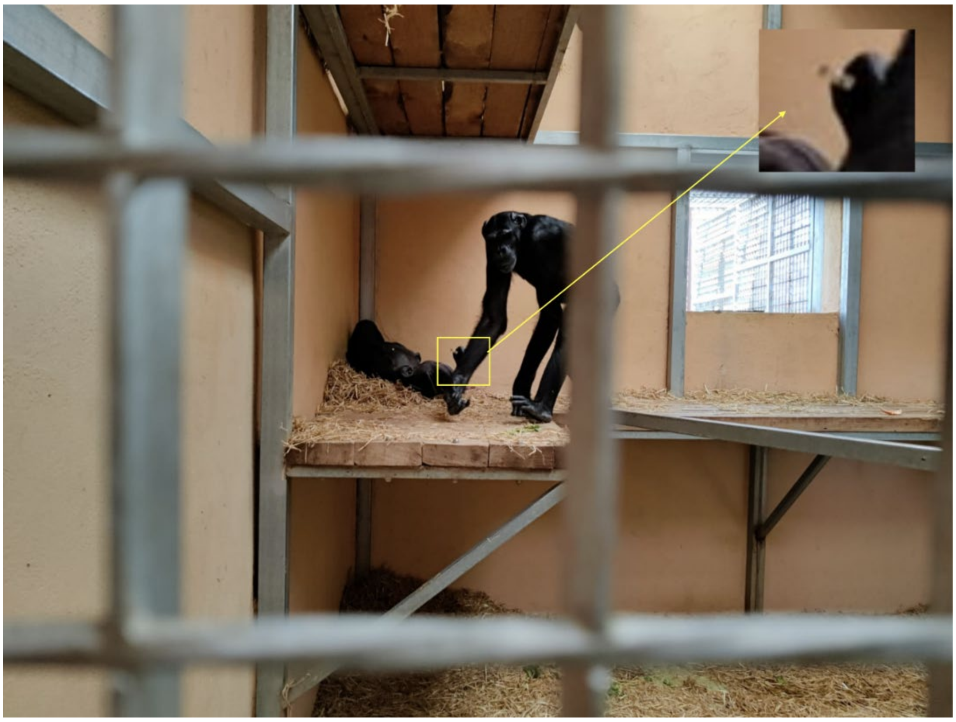

Fig S2. Yvan holding a transparent quartz inside the dormitories.

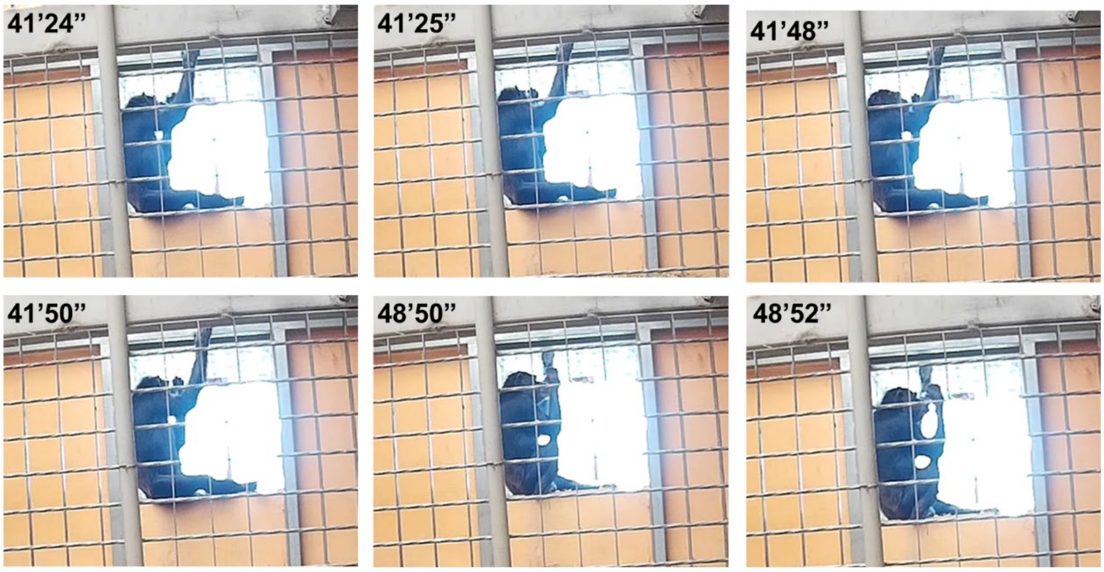

Fig S3. Frames Yvan inspecting the optical properties of a crystal.
Sequential frames showing Yvan at the doorway of the indoor facility carefully examining a transparent quartz crystal taken from a pile of pebbles. He repeatedly brought the crystal close to his eye to inspect its transparency, an activity that lasted for over 15 minutes. Similar inspections were also observed outdoors (Fig. S3).

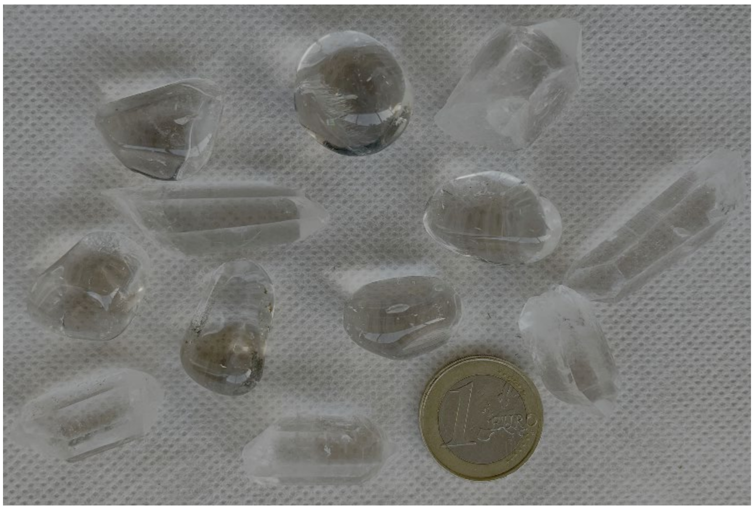

Fig S4. Types of crystals used in Experiment 2.1 in order to explore preference between euhedral and anhedral morphology
